# Perforated duodenal ulcer after Roux-en-Y gastric bypass: an unusual complication

**DOI:** 10.1530/EDM-24-0049

**Published:** 2024-07-13

**Authors:** Ana Munhoz, Cláudia Paiva, Isabel Mesquita, Teresa Correia, Mário Marcos, Jorge Santos, Paulo Soares

**Affiliations:** 1 Unidade Local de Saúde de Santo António - ULSSA, Porto, Portugal; 2 Department of Digestive and Extra-Digestive Surgery, Porto, Portugal; 3 Obesity Treatment Center - Unidade de Tratamento Cirúrgico de Obesidade (UTCO), Porto, Portugal; 4 CAC ICBAS-CHP, Porto, Portugal; 5 I3S, Glycobiology and Cancer Research, Porto, Portugal

**Keywords:** Adult, Male, White, Portugal, Duodenum, Obesity, Gastrointestinal perforation, Unique/unexpected symptoms or presentations of a disease, July, 2024

## Abstract

**Summary:**

Bariatric surgery is increasingly being accepted as a viable treatment for managing the growing obesity epidemic. Roux-en-Y gastric bypass (RYGB) is one of the most commonly performed procedures. Perforated duodenal ulcer following RYGB is a rare condition with a low incidence. We report a case of a patient with a perforated duodenal ulcer post RYGB, and the surgical approach. A 66-year-old man with hypertension and a history of laparoscopic RYGB for class III obesity was admitted to the emergency department with severe epigastric pain radiating to the right side of his abdomen and right shoulder, associated with nausea and vomiting. Computed tomography (CT) showed intraperitoneal free fluid, a thickened wall of the duodenum and free air, duodenal perforation was suspected. The patient underwent exploratory laparoscopy that revealed a perforated duodenal ulcer that was closed with an absorbable barbed suture and omental patch. Perforated ulcers in excluded segments after RYGB are a rare entity with a challenging diagnosis, and clinicians should be aware of and have a low threshold for diagnostic laparoscopy.

**Learning points:**

- Roux-en-Y gastric bypass (RYGB) is one of the most commonly performed procedures in bariatric surgery.
- Perforated ulcers in excluded segments after RYGB are a rare entity with a challenging diagnosis.
- The pathophysiology of this perforation is not clear, but several mechanisms have been proposed. *Helicobacter pylori* has been implicated.
- Clinicians should be aware and have a low threshold for diagnostic laparoscopy for a patient who has acute abdominal pain after RYGB, despite negative diagnostic measures.

## Background

According to the World Health Organization, obesity has nearly tripled since 1975 worldwide. It is reported that more than 1 billion people are obese, affecting morbidity, mortality and quality of life.

Bariatric surgery is increasingly being accepted as a viable treatment for managing the growing obesity epidemic. Surgery can provide a sustainable long-term option for weight loss and control or cure comorbidities (1).

Different surgical techniques have been described to manage obesity, including adjustable gastric banding, sleeve gastrectomy, Roux-en-Y gastric bypass (RYGB), and biliopancreatic diversion with duodenal switch (2, 3).

Roux-en-Y gastric bypass, which combines restriction and malabsorption mechanisms, is one of the most performed procedures, resulting in the exclusion of a stomach remnant, the duodenum and the proximal jejunum.

The most common complications include anastomotic leakage, which occurs in 0.7% to 5% of patients, and stenosis of the gastro-jejunal anastomosis in 3% to 27%. Other complications include bleeding, delayed gastric emptying, marginal ulcer and perforated duodenal ulcer (1, 3).

Marginal ulcer is a known complication which occurs in 4% of the patients, but perforation of a duodenal ulcer is very rare (1, 4). The diagnosis and treatment are challenging due to itsunusual presentation.

We report a case of a patient with a perforated duodenal ulcer after RYGB and the surgical approach.

## Case presentation

A 66-year-old man with hypertension and a history of laparoscopic RYGB for class III obesity performed in 2009 (current body mass index (BMI), 41.5 kg/m^2^ – the BMI and follow-up at the time of the surgery were unknown because the patient was treated in another hospital) was admitted to the emergency department with severe epigastric pain radiating to the right side of his abdomen and right shoulder, associated with nausea and vomiting, for the past 24 h. He denied ingestion of nonsteroidal anti-inflammatory drugs and smoking habits.

Vital signs were stable. The clinical examination revealed a distended abdomen and marked diffuse abdominal tenderness, especially in the upper quadrants.

## Investigation

Laboratory findings were unremarkable.

Chest and abdominal radiography did not demonstrate pneumoperitoneum.

An abdominal ultrasound showed cholelithiasis, gallbladder wall thickening (> 5 mm) and minimal perihepatic-free fluid.

**Figure 1:**
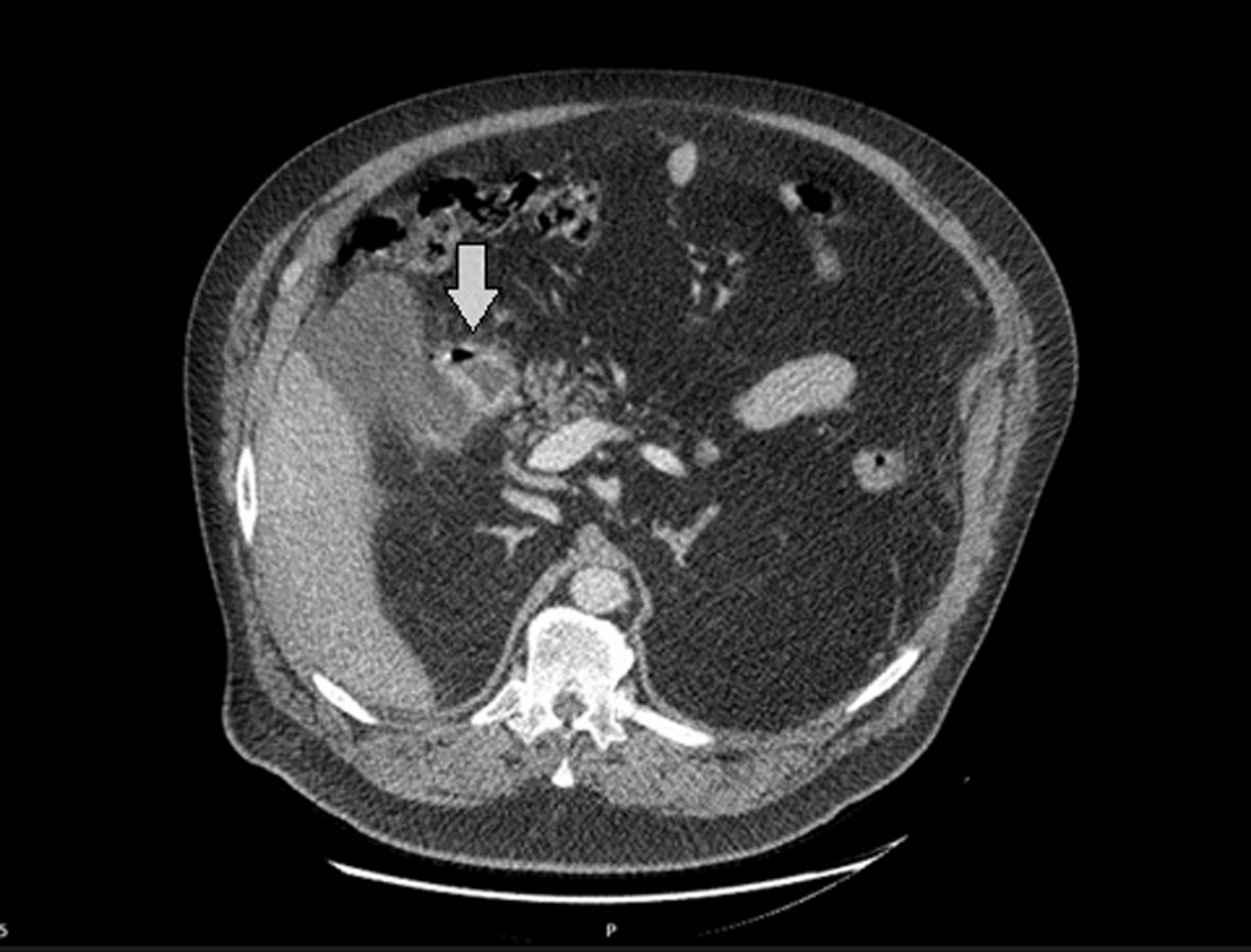
CT showed a thickened wall of the duodenum and free air.

**Figure 2:**
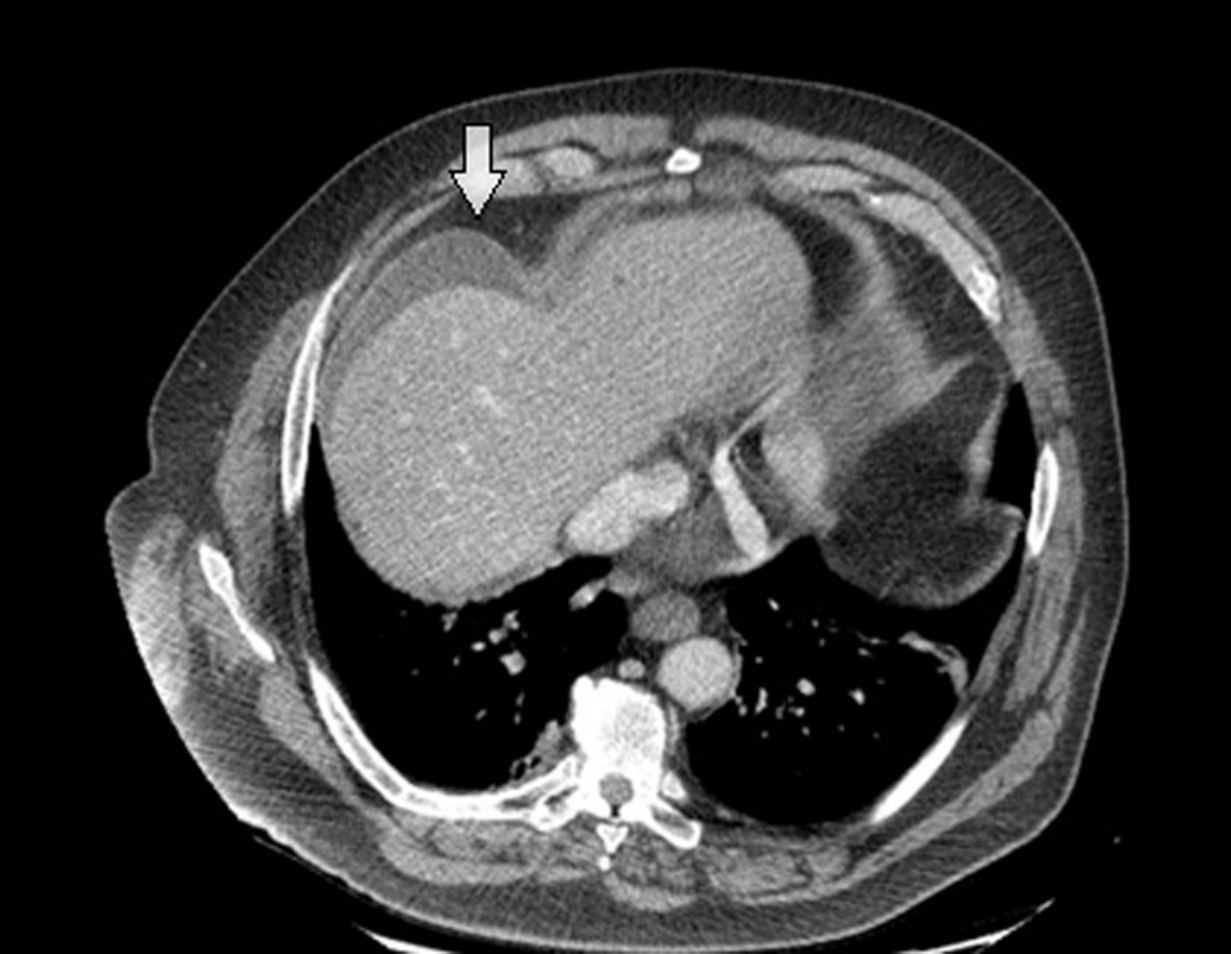
CT showed intraperitoneal free fluid.

The persistent pain led to an abdominal computed tomography (CT) that showed intraperitoneal free fluid, a thickened wall of the duodenum and free air (Figs 1 and 2). Duodenal perforation was suspected.

## Treatment

**Figure 3:**
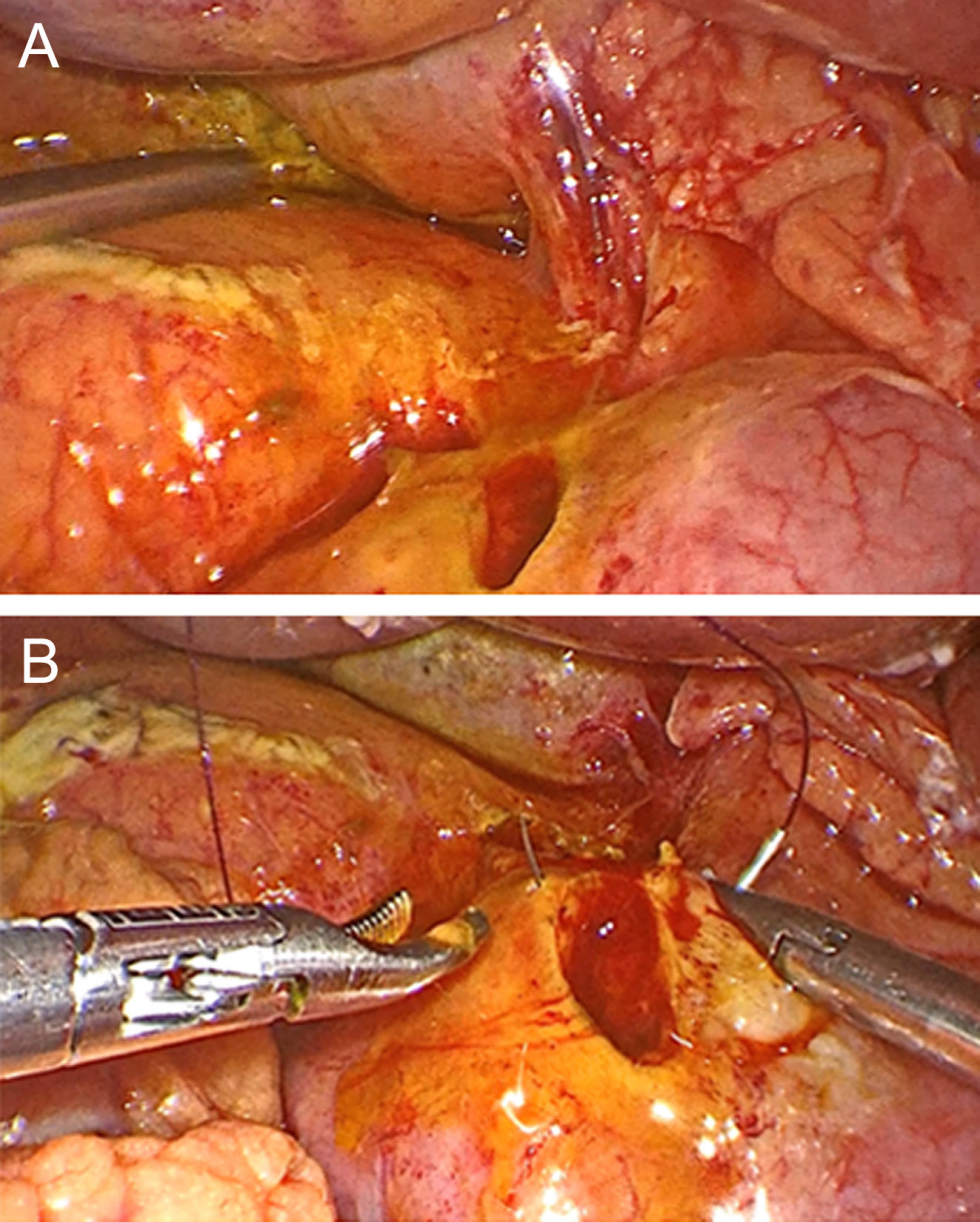
(A and B) Duodenal perforation in the anterior wall.

The patient underwent exploratory laparoscopy that revealed bilious fluid and purulent peritonitis at the right quadrants. At the second portion of the duodenum (D2), a 10 mm perforation was observed in the anterior wall (Fig. 3A and B). The gallbladder was checked without alterations.

The administration of methylene blue ruled out the presence of a gastro-gastric fistula. The ulcer was closed with an absorbable barbed suture and an omental patch.

## Outcome and follow-up

**Figure 4:**
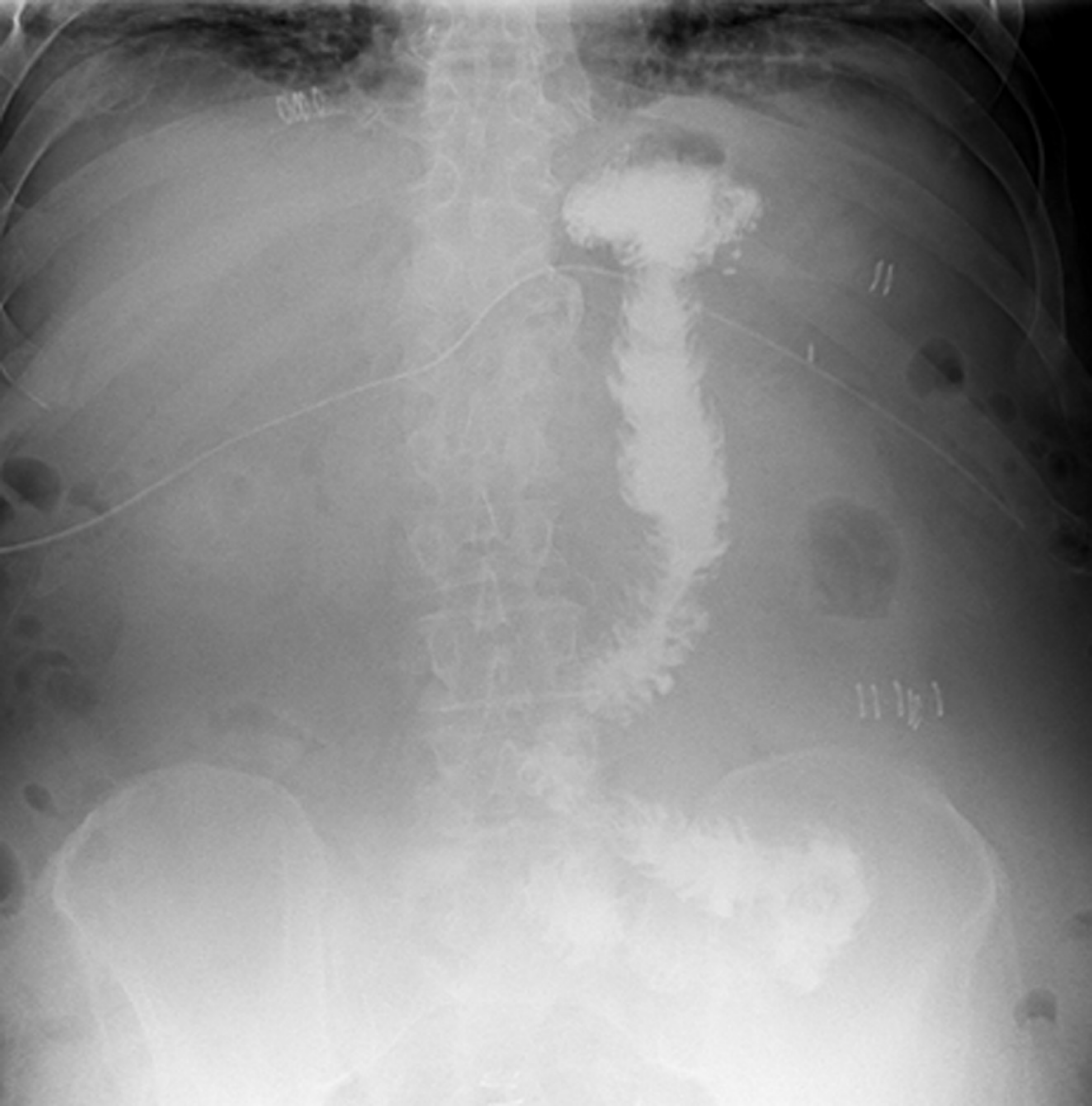
Upper gastrointestinal series with no leak, excluding a gastro-gastric fistula.

The postoperative course was uneventful. An upper gastrointestinal series (UGI) 5 days after the intervention demonstrated timely contrast passage with no leak, excluding a gastro-gastric fistula (Fig. 4).

Intravenous piperacillin–tazobactam and fluconazole were continued for 7 days. Feeding was started on the second postoperative day and the patient was discharged on day 7 without any complications. *Helicobacter pylori* (*H. pylori*) eradication and lifelong proton pump inhibitors were prescribed.

## Discussion

Perforated duodenal ulcer following RYGB is a rare condition with a low incidence, that is difficult to diagnose due to the nonspecific clinical findings. The excluded digestive segments exhibit a different behaviour complicating diagnosis, treatment and follow-up (2).

Given the anatomical characteristics of the gastric remnant, conventional signs of perforation, such as free air, might not always be present. Usually, if free air is present, the lesion in the excluded segment is accompanied by another problem, like obstruction below or at the jejuno-jejunostomy or a gastro-gastric fistula, which we ruled out in our patient with an upper gastrointestinal series (2).

CT scan is the most accurate exam to diagnose a perforation of the excluded stomach or biliopancreatic limb, even though imaging studies can fail to identify a small duodenal perforation because of the lack of free air and no contrast extravasation. In addition, a CT scan will help identify other possible causes of the acute surgical abdomen in a patient after RYGB such as internal herniation (5).

The pathophysiology of this perforation is not clear, but several mechanisms have been proposed. *H. pylori* has been implicated in the formation of ulcers by weakening the mucosal protective barrier (5). Other risk factors described are smoking, ingestion of nonsteroidal anti-inflammatory drugs, and alcohol consumption.

Another possible mechanism described in the literature suggests that it might be caused by unbuffered acid in the bypassed segment, because there was no contact between acid production in the remnant stomach and ingested food (4, 5, 6).

The etiology of the ulcer in our case is unknown because none of these known risk factors were present.

Surgical management of perforated duodenal ulcers includes closure of the defect or resection of the remnant part of the stomach.

Our patient underwent laparoscopic closure of the ulcer and omental patch, without complications.

Resection of the gastric remnant would lead to a decrease in acid production, avoid the formation of a gastro-gastric fistula and eliminates the difficulties in accessing the gastric remnant, but it is not without consequences, exposing the patients to greater surgical trauma and an increased risk for surgical complications and metabolic consequences, such as vitamin B12 deficiency (4, 5).

Managing the perforation, the treatment should include risk factors control, like eradication of *H. pylori* and lifetime proton pump inhibitor, to reduce recurrence.

## Conclusion

Perforated ulcers in excluded digestive segments after RYGB are a rare entity with a challenging diagnosis. Clinicians should be aware that a CT scan is often essential for a patient who has acute abdominal pain after bariatric surgery, and has a low threshold for diagnostic laparoscopy despite negative diagnostic measures. Early surgical exploration remains the treatment of choice.

## Declaration of interest

The authors declare that there is no conflict of interest that could be perceived as prejudicing the impartiality of the study reported.

## Funding

This study did not receive any specific grant from any funding agency in the public, commercial or not-for-profit sector.

## Patient consent

Written informed consent has been obtained from the patient for publication of this case report.

## Author contribution statement

AM wrote the manuscript, which was reviewed by IM and PS, who also edited and finalized the version of the manuscript. The remaining authors were involved in patient management.

## References

[1] Ma IT & Madura JA II. Gastrointestinal complications after bariatric surgery. Gastroenterology and Hepatology 2015 11 526–535. 27118949 PMC4843041

[2] Pohl D Schmutz G Plitzko G Kroll D Nett P & Borbely Y. Perforated duodenal ulcers after Roux-Y gastric bypass. American Journal of Emergency Medicine 2018 36 1525.e1–1525.e3. (10.1016/j.ajem.2018.04.057) 29716802

[3] Cuadrado-Franco D & Díaz S. Perforated duodenal ulcer after laparoscopic Roux-Y gastric bypass. Case report. Iatreia 2021 34 365–369. (10.17533/udea.iatreia.113)

[4] Alotbi R Allam HM Almaghrabi AH Ghazi AA & Alzahrani AG. Perforated duodenal ulcer following Roux-en-Y gastric bypass (RYGB): case report and literature review. International Journal of Medical Research Professionals 2017 3 292–294. (10.21276/ijmrp.2017.3.6.058)

[5] Iskandar ME Chory FM Goodman ER & Surick BG. Diagnosis and management of perforated duodenal ulcers following Roux-en-Y gastric bypass: a report of two cases and a review of the literature. Case Reports in Surgery 2015 2015 1–4 (10.1155/2015/353468). PMC4408622 25949843

[6] Mittermair R & Renz O. An unusual complication of gastric bypass: perforated duodenal ulcer. Obesity Surgery 2007 17 701–703. (10.1007/s11695-007-9122-2) 17658034

